# Whole-Genome Sequence of *Bradyrhizobium elkanii* Strain UASWS1015, a Highly Ammonia-Tolerant Nitrifying Bacterium

**DOI:** 10.1128/genomeA.00111-16

**Published:** 2016-03-10

**Authors:** Julien Crovadore, Gautier Calmin, Bastien Cochard, Romain Chablais, François Lefort

**Affiliations:** a Plants and Pathogens Group, Research Institute Land Nature and Environment, hepia, HES-SO University of Applied Sciences and Arts Western Switzerland, Geneva, Switzerland; b Faculty of Engineering and Architecture, HES-SO University of Applied Sciences and Arts Western Switzerland, Delémont, Switzerland

## Abstract

*Bradyrhizobium elkanii* UASWS1015 was isolated from a sewage plant in Switzerland. Its genome indicates that it is fully equipped for ammonia assimilation and aromatic compound degradation, and it displays a large type IV secretion system, which characterizes plant-associated microbes. Totally deprived of toxins, it could be considered for agricultural and environmental uses.

## GENOME ANNOUNCEMENT

Bradyrhizobia establish symbiosis with legumes by forming root or stem nodules, and some can be photosynthetic. *Bradyrhizobium elkanii* (1), an aerobic, motile, and Gram-negative rod, does not form spores and can be found in a free-living state or as a plant symbiont. This species is also known for producing bioemulsifiers (2) and is used in agriculture (3).

Strain UASWS1015 was isolated when selecting for highly ammonia-tolerant nitrifying bacteria from a sewage plant, and it was first identified as *B. elkanii* by 16S sequencing. Genomic DNA was extracted from a pure axenic culture, according to an adapted protocol (4). Libraries were made using the Nextera XT kit (Illumina, USA). Whole-genome shotgun (WGS) sequencing was performed within one Illumina MiSeq run at 2 × 250-bp read length and yielded 187 contigs, providing 72.3× genome coverage, for a genome total length of 7,820,754 bp, G+C content of 64.6%, and a scaffold *N*_50_ value of 315,183 bp. Among the 10 genomes available in NCBI genome database for this species, this strain displays the highest G+C content and is genetically close to two strains, *B. elkanii* USDA 3259 and USDA 3254, while its genome size is smaller. Raw reads were trimmed with FastQC (http://www.bioinformatics.babraham.ac.uk/projects/fastqc) and assembled with the SPAdes genome assembler 3.6.1 (5). The resulting contigs of the genome assembly were arranged with BioEdit (6) and analyzed with QUAST (7). *In silico* screening with PlasmidFinder (8) did not identify any circular or integrated plasmid genome. Automated gene annotation was carried out using the NCBI Prokaryotic Genome Automatic Annotation Pipeline PGAAP (9) and reviewed with RAST version 2.0 (10). It allowed for the identification of 7,285 genes distributed in 6,823 coding sequences (CDSs), 398 pseudogenes, 64 rRNA genes (5S, 16S, 23S, tRNAs, and 1 noncoding RNA [ncRNA]), and 26 frameshifted genes, while RAST analysis identified 7,658 CDSs. No complete transposon or phages were found to be integrated in the genome. Toxin, superantigen, virulence, and disease genes are absent, which allows this bacterium to be considered a biological fertilizer. While some *B. elkanii* strains harbor type III and type IV secretion systems (11), strain UASWS1015 possesses a large type IV secretion system, known in many plant-associated microbes, which is composed of 29 genes for Vir proteins (12). Additionally, it is equipped with 15 genes for bacteriocin and antimicrobial synthesis and 112 genes involved in antibiotic, multidrug, and heavy-metal resistance. The bacterium is fully equipped for ammonia assimilation. Additionally, 115 genes are involved in numerous pathways of aromatic compound degradation, offering a possible role for soil depollution. It also contains 4 genes coding for a photosystem reaction center, and while a few genes for nodulation (*nod* and *noIO*) are present, the most important *nod* genes, A, B, and C (13), are absent, making this bacterium possibly unable to nodulate, a situation which has already been described for symbiotic photosynthetic *Bradyrhizobium* species (14). This unusual genome of *B. elkanii* would contribute to a better knowledge of this species, and ongoing works confirm that it might be usable in agriculture, wastewater, and contaminated soil management (unpublished data).

### Nucleotide sequence accession numbers.

This WGS project was deposited at DDBJ/EMBL/GenBank under the accession no. JXOF00000000. The version described in this paper is the first version, JXOF00000000.1. The 187 contigs have been deposited under the accession no. JXOF01000001 to JXOF01000187.
